# Social and Moral Practices of the Organizations and Employee-Based Brand Equity: Female Digital Labor Perspective

**DOI:** 10.3389/fpsyg.2022.910483

**Published:** 2022-06-03

**Authors:** Sha Hu

**Affiliations:** College of Literature, Beijing Language and Culture University, Beijing, China

**Keywords:** socially responsible management, organizational morality, employee vitality, employees’ moral engagement, employee-based brand equity

## Abstract

This study investigates the role of socially responsible management as a significant determining factor for employees’ morale engagement (EME), employee vitality (EV), and employee-based brand equity (EBBE). Human resource management policies and strategies are important for addressing the interests of the employees and boosting the overall effectiveness of the organization. To examine this, this study analyzes the role of socially responsible management and organizational morality on EME with the mediation of EV. Also, the study examines the role of EME in EBBE. To conduct this study, the data were obtained from 310 female employees working in software houses from home in China. The sampling technique used in the study is purposive sampling. A partial least square structural equation modeling technique is used to analyze the data of the study. The study found that socially responsible management has a positive effect on both EME and EV. The study also reveals that organizational morality has a positive impact on EME and consequently impacts the EBBE in a positive way. The results of the mediation analysis show that EV mediates the relationship between socially responsible management and EME. The study provides practical implications, explaining the strategies and policies that can be adopted by the HR department of the organization to boost employee interests. The study also provides some limitations and future recommendations, such as sample size, the context of the study, and adding new variables to the existing framework.

## Introduction

Employees are the most important stakeholders in a service brand. They work as internal ambassadors for the branding of the organization. Branding of service firms is largely dependent on brand commitment. Brand commitment is developed through the experience and contacts of the employees in an organization. Such experience and contacts of the employees also shape the employees’ personal views, emotions, and behaviors (Brexendorf and Kernstock, 2007). These experiences also led to the development of employee-based brand equity (EBBE), which was proposed by King et al. (2013). EBBE is defined as employees’ constructive brand behaviors that are derived from their brand knowledge. These behaviors are consistent with the brand identity’s desired behavior. According to literature, a company’s EBBE is shaped through reductions in recruitment expenditures, enhancement of employees’ performance, and the generation of more revenue. Therefore, EBBE is an essential issue for further research in the disciplines of branding and internal brand management (Lee et al., 2019).

Employees are the internal stakeholders of a company due to the fact that they take part in shaping the company’s values. This is a crucial element in internal branding as the continuous transmission of the brand message to consumers is improbable without it (Helm et al., 2016). Internal branding progress depends on internal stakeholders’ synchronization with the company’s values. It also helps in assessing the products and consumers’ behaviors. Consumers’ experience with brand messages remains ineffective without employees’ congruence with the organization’s values (Du Preez et al., 2017). These organizational values to develop EBBE come from certain organizational management activities. In this study, it is assumed that EBBE may be developed through certain practices such as socially responsible management, organizational morality (OM), employee vitality (EV), and employees’ moral engagement (EME). These have been explored in different ways in the past, but their possible role in EBBE is proposed in this study.

Employee engagement is considered as a contributor to EBBE. It refers to a person’s moral connection to the company they work for. This is demonstrated by the worker’s commitment, outreach, discretionary endeavors, expertise, abilities, and competencies (Tamunomiebi et al., 2020). Morally engaged employees are those who function with enthusiasm and have a greater bond with their employer. Employees are responsible for driving creativity and propelling the firm forward (Tamunomiebi et al., 2020). Engaged employees who perform efficiently are more creative than others. They are more inclined toward their organizations and have a desire to continue their jobs in their organizations. They have higher levels of personal wellbeing and consider their employment to be more reasonable than everyone else’s (Jiang and Men, 2015).

Engaged employees remain in the organization longer and continue to develop new and better approaches to provide considerable value to the company. Employee engagement entails putting in physical, mental, and psychological effort at job at the same time (Kahn, 1990). Employee engagement gets a moral support when employees start believing that their companies have started paying attention to economic as well as environmental issues. Since such socially responsible management efforts are virtuous, they can help them find significance in their occupations and in the functioning of the company (Afsar et al., 2020). Employee engagement is difficult to achieve, yet it is incredibly valuable for businesses. For example, in the United States, less than 33% of employees think that they are engaged in their work, and the scenario is comparable with many other countries (Farndale and Murrer, 2015).

It is becoming equally important to consider what motivates employees at their jobs. The managers are looking for new and effective ways to boost employee moral engagement (Knight et al., 2017). Although a number of individual and organizational factors have been recognized as having an impact on employee moral engagement, the impact of socially responsible management has not been studied before on EME (Afsar et al., 2020). Therefore, this study tries to fill this gap. One of the most significant company resources is the human aspect (i.e., the employee group). As employees of organizations are treated under human resource management (HRM), it becomes an important component in the organizational brand equity dimension. HRM is commonly regarded as a critical component in attaining a competitive edge at the organizational level. HRM has been shown to have a positive impact on organizational performance and efficiency by influencing employee behavior and work attitudes (Voorde and Beijer, 2015).

Various investigations have looked into the influence of HR practices on employee work outcomes during the past few decades. HR practices were found to be positively related to employee behavior and work attitudes in the majority of these investigations (Vanhala and Ritala, 2016). There are certain other HR practices which may influence the EME, e.g., socially responsible management (SRM). Shen and Jiuhua Zhu (2011) popularized the notion of SRM. This SRM includes recruitment efforts and the preservation of socially responsible workers. SRM offers training on corporate social responsibility (CSR) and considers employees’ performance outcomes when contemplating promotional offers, performance appraisals, and rewards. SRM also helps in providing excellent wages and working conditions to the employees (Shen and Benson, 2016).

Internal stakeholders are more likely to approve this form of HRM practice (e.g., employee groups). A lot of studies have also looked into the impact of such HR practices on employee performance, which could lead to the development of EBBE (Newman et al., 2016; Shen and Benson, 2016). As indicated by Abdelmotaleb and Saha (2020), SRM is the type of CSR practices, which are directed toward the employees of organizations. It is obvious that such management practices lead to manage employees’ performance at an organizational level. The majority of the current investigations on socially responsible management have focused on determining how these practices affect the attitudes of external stakeholders, including consumers, authorities, and commodity markets. This kind of management of the company and its impact on the company’s performance remain largely unknown (del-Castillo-Feito et al., 2022).

Enterprises can use a variety of socially responsible management approaches and give vital significance to employees who engage in organizational productivity. Enterprises implement socially responsible practices that influence the financial planning process, knowledge, and culture. Using social responsibility activities, like creating fluid interactions, helps the organization develop trust and engage employees effectively (Blanco-Gonzalez et al., 2020). Another aspect of EME is influenced by OM. Morality is an attribute that encourages an employee to be on time when expected. This is possibly achieved through OM, which has an impact on every employee.

Morally focused organizations do not take part in any of the corrupt practices. Such organizations do not get involved in kickbacks after the contracts are awarded to stakeholders. Window dressing, accounting tricks, and off-financial statement activities are discouraged by morally focused organizations to maintain a favorable and respectable impression on the public. Employee and workplace success are admirable when it comes to ethical integrity and ethical values (Sani, 2018). Morality influences how employees perceive their work and organize their presence or position in the company. The virtue of morality encourages the employees to not violate the norms and practices during a lack of monetary compensation. It is crucial to remember how workplace behavioral morals and conduct have an impact on employees’ personal bonding with their company and the activities they take part in to support the company’s success.

Employees show more involvement if their workplace morals and professional ethics are upheld. They show commitment, devotion, passion, responsibility, and a focus on results and efficiency (Tamunomiebi et al., 2020). Organizational morality is characterized as the norms and requirements of conduct for an organization. It requires employees to perform in accordance with the established behavioral practices and regulations in order to enhance the organization’s overall good. Morality claims all employees’ lifestyles more than the law does, and it takes precedence over personal employee interests. Workers have basic responsibilities and obligations to the company. The failure of workers to follow the correct behavior in the workplace necessitates the establishment of a morality at work framework (Sani, 2018; Tamunomiebi et al., 2020).

There has been a dearth of research regarding OM and EME (Tamunomiebi et al., 2020), which paves the way to explore the gap. Therefore, this research tried to assess this possible relationship. There are certain other factors, which may have an impact on employees’ engagement leading, to the development of EBBE, e.g., physical strength of the employees, such as vitality. Scholars have been paying more attention to employee sentiments of vitality at work in recent years. It is crucial to understand what elements influence EV at work since a vitalized person has better mental and physical health. Health-related attributes are key factors in dealing with and overcoming organizational difficulties and challenges. Greater efficiencies in dealing with work challenges are related to a greater level of vitality.

Workers that are more vitalized have stronger emotional energy and a stronger degree of mental liveliness, making them more proactive at work (Jahanshahi et al., 2019). This helps employees and their colleagues in having a more pleasant work atmosphere. There are various empirical investigations that support the relevance of vitality to jobs. Vitalized employees are far more efficient, innovative, and motivated to work and have a favorable impact on customer satisfaction. Employees who are less energized, on the contrary, are likely to perform poorly. In general, the low extent of vitality inside an organization may lead to higher levels of long-term desire to quit (Tummers et al., 2018; Jahanshahi et al., 2019).

In recent times, research has been conducted on the impact of employees’ vitality in the context of physically dangerous workplaces like war zones, but no one has tried to explore the impact of employees’ vitality on the engagement of employees. This also posed a gap in research studies. It allowed us to evaluate the mediating role of EV between socially responsible management, OM, EME, and EBBE. This research tries to combine all these aspects of developing EBBE for organizations. For this purpose, this study looks into the possible direct associations between SRM and organizational moral engagement, leading to EBBE.

## Theoretical Support and Hypothesis Development

This study is supported by virtue ethics theory (Kaptein, 2008), which supports the notion of OM. Organizational morality is a set of ethical virtues and the ethical culture of the organizations, which can influence the employees regarding their moral engagement with the organization and ultimately influence EBBE. Even though there has been a gradual growth in interest in the issue, empirical studies of virtue ethics within business ethics or morality research have been rather restricted. Some more comprehensive utilization of the theory in actual conditions is urgently required (Dawson, 2015). This theory provides the basis for shaping organizational ethical virtues. These virtues have been about continuously practicing, improving, and revamping OM from an organizational perspective, not only listing features or traits (Chun, 2005).

The circumstances, customs, and practices of organizational conduct shaping the ethically sustainable behavior of employees are referred to as OM. Using virtue-ethics theory, Kaptein (2008) claims that an organization’s morality is based on organizational ethical virtues that encourage individuals to behave ethically. Socially responsible management in organizations is a type of HRM, and to obtain competitive advantage of any firm, HRM plays a key role (Shen and Jiuhua Zhu, 2011). It has an impact on organizational performance through influencing staff attitudes and behaviors, such as EME. Therefore, such socially responsible management practices get support from attribution theory (Shen and Jiuhua Zhu, 2011).

This study also gets support from two more underlying theories in the context of socially responsible management, OM, and EME for developing EBBE. Social exchange theory and equity theory have provided foundations to similar studies in past (Tamunomiebi et al., 2020). Social exchange theory and Adams’ equity theory are used to explain the link between corporate morality and EME (Adams, 1963; Cook et al., 2013). Improved engagement, from these beliefs, can only be achieved if the social exchange among firms and workers is equal and fair. Workers are more willing to connect to the business with more moral engagement if they believe that the organization is committed to a code of ethics (morality), ethical perceptions, and behaviors.

Furthermore, management serves as a role model for employees, helping them to promote company goals and objectives by demonstrating excellent ethical behavior. As a result, OM is capable of having an impact on employee moral involvement. Employees and organizational socially responsible management would have more confidence in such an ethical environment, which would strengthen their commitment to their organization and work (Tamunomiebi et al., 2020). This study also gets a theoretical support from the cognitive evaluation theory of Deci and Ryan (1985), according to which employees get insights from their surroundings and, based on this information, mold their energy level. Such energy levels are important for their vitality to work in organizations. Similar studies like Huang and Chen (2021) got support for the backing of EV as a mediator. So, this study also looked into the mediating role of employee loyalty based on this theory.

### Socially Responsible Management, Employee Vitality, and Employees’ Moral Engagement

Socially responsible management is characterized as CSR oriented toward employees. Several studies have been undertaken over the last decade to determine the connection between employee impressions of SRM, employee behaviors, and work practices (Shen and Benson, 2016). Shen and Zhang (2019), for example, looked into the relationship between SRM and promotion of employees for the perception of CSR. Perception of SRM among employees and CSR showed a linkage among themselves (Shen and Zhang, 2019). Shen and Jiuhua Zhu (2011) found that SRM had a substantial positive relationship with employee commitment in a sample of 784 managers and employees in two manufacturing enterprises in China.

Kundu and Gahlawat (2015) revealed that perceptions of employees toward SRM were linked positively with job satisfaction in a study of 563 workers in different industries in India. Job satisfaction seemed to have a full mediating influence on the association between SRM and employee turnover intention, according to the findings of their study. Furthermore, through the mediation of employees’ organizational identity, Shen and Benson (2016) discovered that SRM perceptions interacted with perceived organizational support to influence employee task performance on extra-role assisting behavior. Perceptions of employees about SRM were found to be a significant antecedent of work attitudes and behaviors in several studies (Abdelmotaleb and Saha, 2020).

Understanding the antecedents and determinants of employee engagement, which include socially responsible management, has been a major focus of scholarly work on employee engagement taken from human resources, strategic planning, and marketing publications (Stephanie and Gustomo, 2015). A research citing a lot of investigations has identified that focusing on CSR practices in the form of socially responsible management of employees has produced significant results (Duthler and Dhanesh, 2018). Such practices of SRM could be utilized further to evaluate the behaviors of employees such as their vitality at workplace and moral engagement with their organizations. It is also assumed that SRM could influence EME, which could develop EBBE in the organizations as an internal management approach. Therefore, based on this supporting literature, this study proposes the following hypotheses:

**H_1_:** Socially responsible management has a positive effect on employee’s moral engagement.

**H_3_:** Socially responsible management positively affects employee vitality.

### Organizational Morality, Employee Vitality, and Employees’ Moral Engagement

The extent to which organizations are perceived by their respective employees to maintain universal moral ideals of integrity, honesty, and reliability is referred to as OM (Abdelmotaleb and Saha, 2020). Previous study has focused on morality as a collective attribute (Shadnam et al., 2020). Workers are extremely driven to accomplish what is moral, according to previous studies, and they prefer to associate with moral groups and organizations (Ellemers and Van der Toorn, 2015). According to Ellemers and Van der Toorn (2015), firms should work to improve their moral image in order to recruit, inspire, and retain personnel. A few studies have looked into the relationship between organizational and cultural qualities, as well as employee perceptions of OM.

The normal rules of conduct and behaviors anticipated for employees within the organization are known as OM or workplace ethics. At work, the organization and its employees are required to display morals and behaviors that are sufficient to meet the needs of people (Kaptein, 2008). The relationship between organizational culture and perceived OM has been explored in the past, and culturally specific features were found to have a significant impact on employees’ perceptions of OM (Racelis, 2010). Employee impressions of a firm’s CSR initiatives on OM were investigated by Ellemers and Van der Toorn (2015). They discovered that corporate ethical activity in the context of ecological management and community engagement had a beneficial impact on OM perception.

According to Cohen et al. (2014), OM or personal integrity are standards of normative ethics, damaging acts that are broadly defined as markers of moral or ethical attitude/behavior. Organizational morality, in this context, refers to the appropriate standards defining what is proper and improper in the workplace, which employees of the organization should follow in order to run successful businesses. Every employee in the organization has basic tasks, duties, and obligations to the company. There are a few things that employees have to do and many others that they have to avoid. This indicates that organizations have a moral worth that governs how employees perform tasks and roles. This also includes employee-to-employee, employee-to-management, and management-to-shareholder interactions, as well as the firm’s ties with customers, suppliers, and dealers, as well as the general public. All of these organizational interactions are governed by workplace ethics (Hough et al., 2015).

Employee moral engagement, which is defined as employees’ emotional attachment to the company and their willingness to contribute their fair share to the company’s success, could be influenced by a variety of factors. Employee engagement is strongly linked to OM and organizational ethics. Employees would be more engaged morally if the company or workplace had a clear moral and ethical code (Kaptein, 2013). Employee moral involvement is strongly correlated with a decent code of practice and OM in the workplace. Studies have shown that this is true (Kaptein, 2013; Cohen et al., 2014). Employee vitality pertains to an employee’s state of positive alertness and energy in relation to his or her teammates at work in this study. Vitality has long been seen as a key indicator of an employee’s psychological and physical wellbeing (Garg and Sarkar, 2020).

Elevated levels of independence, the ability to participate in organizational decision-making activity, and good quality cooperation were discovered to be essential organizational management practices for boosting EV in this line of research (Tummers et al., 2015). Employees who are intrinsically motivated have a greater sense of vitality in the profession. As a result, they are more engaged at work and more innovative at work. Organizational morality has been studied in the context of work engagement by the employees, which allowed us to find associations between employees’ attributes such as EV and EME with the organizations. So, this study postulates the following hypotheses:

**H_2_:** Organizational morality positively influences employees’ moral engagement.

**H_4_:** Organizational morality has a positive effect on employee vitality.

### Mediating Role of Employee Vitality

Perceptions of vitality relate to an employee’s condition of pleasant alertness and energy in relation to his or her teammates at work in this study. Employee vitality has long been seen as a key indicator of an employee’s psychological and physical wellbeing (Dutton and Heaphy, 2003). Numerous external and internal elements are essential to improve such aspects in the organization, according to the literature (Garg and Sarkar, 2020). Effective interpersonal interactions among colleagues are crucial for boosting vitality at work (Carmeli, 2009). According to the findings of a recent study conducted with employees of a Dutch dairy firm, having a decent work–life harmony and leading a healthy lifestyle improves the feelings of vitality in the workplace (Tummers et al., 2015).

Certain HRM techniques were discovered to be crucial for boosting EV throughout this line of research: Strong levels of confidence, the ability to participate in corporate decision-making, and high-quality teamwork are all desirable (Scheppingen et al., 2014; Tummers et al., 2018). Employees that are intrinsically motivated have a greater sense of vitality in the organization. As a result, they are more engaged at work and more innovative in their work (Strijk et al., 2012; Xie et al., 2020). Jansen (2004) discovered that increased levels of vitality among employees enhance the organizational transition process in this regard. Moreover, vitalized employees have greater mental fortitude in the workplace, which is a key component in dealing with obstacles and challenges.

Previous studies have mostly concentrated on exploring the implications of EV (Tummers et al., 2018). Tummers et al. (2018) looked at how work factors affect EV. The key job features, namely, management assignment transmission and work engagement, were found to be positively associated with EV at the workplace. Despite the fact that positive employee affective experiences in the workplace are thought to lead to increased EV (Ryan et al., 2010), little attention has been paid to its role as a mediator between specific management elements of employees in businesses. Very limited research in the past has been carried out to find the mediating role of EV but proved its significance among certain organizational factors (Kark and Carmeli, 2009). Therefore, the authors suggested that it could mediate the relationship between socially responsible management, OM, EME, and EBBE.

**H_5_:** Employee vitality mediates the relationship between socially responsible management and employees’ moral engagement.

**H_6_:** Employee vitality mediates the relationship between OM and employee’s moral engagement.

### Employees’ Moral Engagement and Employee-Based Brand Equity

Employees who are engaged have a strong relationship with their firm and invest not only in their individual responsibilities but also in the organization as a whole. Over time, those who are involved with the institutions with which they are affiliated or the firm for which they work outperform their colleagues in regards to productivity, and they constantly function as company advocates (Sendawula et al., 2018). Employee engagement and commitment to the company would always increase performance and greatly enhance the company’s profits. Individuals who are totally engaged in work have higher self-efficacy and have a beneficial impact on their health, which leads to the enhanced active support for business. Employees in this group consider themselves to be actual participants in the organization (Pandita and Ray, 2018).

Employee engagement in organizations is influenced by a number of things. The job nature, a job with clear purpose and meaning, career progression, reward and recognition opportunities at work, constructing appreciation and confident connections, open lines of communication processes that encourage governance, and coaching opportunities are all examples of these considerations. The inference is that for every engagement, there is still a key cause or set of reasons that either excite or demoralize employees, resulting in a lack of engagement (Sendawula et al., 2018). According to Jena et al. (2018), who performed a study in various countries and across industry sectors, passionate and dedicated employees are in the minority. State participation, once again, boosts productivity and leads to company profitability.

It is established that morally engaged employees with their organizations have impacts on the organizational performance in the form of turnovers and productivity. The leading concern of this research lies in the development of EBBE. It is also well established that more engaged employees with their organizations have the ability to perform well in any given circumstance. While EBBE is also a dimension of internal brand management within organizations, it is believed that employees who are morally engaged to their organizations could provide a tool of internal management for developing EBBE. This has been developed on the notion derived from certain previous investigations (Gupta and Shaheen, 2018; Boukis and Christodoulides, 2020). Therefore, this study proposes the following hypothesis:

**H_7_:** Employees’ moral engagement positively affects employee-based brand equity.

**FIGURE 1:**
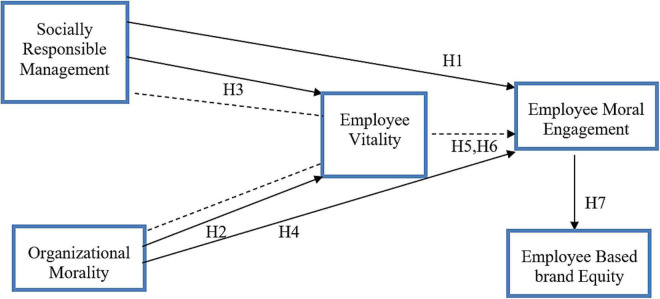
Theoretical framework.

Based on the above literature and hypothesis, the conceptual model (Figure 1) has been established.

## Methodology

This study used a deductive approach to examine the hypotheses of this study. The quantitative research design was chosen to carry out this study. The hypotheses were developed to examine the influence of independent variables on outcome variables. Reliable results were obtained as the study used a quantitative research design, and this research design was found to be beneficial in eliminating the biases. Data collection was carried out using a self-administrated survey. Data rationality was obtained by keeping the questionnaire precise and clear so that the respondents could conveniently fill it out. Female employees of the software houses were the target population of the study. The female employees were specifically targeted to understand the stance of female employees regarding the social and moral practices observed in the organizations considering their gender and to what extent do these practices contribute to the EV and their engagement in their jobs. For this purpose, the female employees who were working from home were approached to participate in the survey. The desirability bias of the participants has been controlled through adopting the current methodology. The authors met the potential female employees working online from home before taking appointments to explain the study and taking permission from the potential participants to participate. The language of the questionnaire was English, which is an international language and understandable in China. The questionnaire was distributed with a cover letter where the purpose of the study was explained, and the method to solve the questionnaire was explained to avoid misunderstandings in the survey. The participants have been assured to keep the responses confidential, and they will be used for the research purpose only. Moreover, the responses obtained from the questionnaire were natural as the respondents were informed that there were no wrong or right answers.

A careful sample estimate of 500 was made based on the study conducted by Wolf et al. (2013), who have investigated what parameters affect the statistical power, parameter estimate biases, and overall statistical analysis and found that a sample size between 30 and 460 cases is found to produce meaningful trends and patterns among the parameters using structural equation modeling (SEM). This study used the time lag method for data collection (Bashir et al., 2021). The questionnaire was divided into two waves. In the first wave (W-1), the data on the independent variables of the study were collected by distributing 500 questionnaires. The questionnaires were collected after 2 weeks, and the number of questionnaires received was 412. With the gap of 40 days, the second wave (W-2) of the data collection was conducted by distributing 412 questionnaires to the respondents of the first wave. After a time period of 2 weeks, 337 filled questionnaires were obtained, but the usable questionnaires were 310, which met the sample size criteria mentioned in literature (Wolf et al., 2013). The rest of the questionnaires were discarded as they were improper and incomplete. The rate of usable response was 81.58%. Statistical software was used for data analysis.

The target population of the study was female employees working in software houses from home; therefore, the data were collected from the target population. Purposive sampling was used to select the sample from the whole population. This type of sampling technique is suitable for this study because it requires less time and is less expensive to acquire data from the most relevant respondents (Etikan et al., 2015), because the researchers needed the female employees working online from home. Therefore, this purposive technique for selecting the sample was the most appropriate. This study has a sample size of 310. The unit of analysis for this study was the female employees working in software houses from home in China; therefore, the data were collected from the target population.

### Measurement

A 5-point Likert scale was used to obtain data for all the variables. The scales were adapted from the past researches (Carmeli and Spreitzer, 2009; King and Grace, 2010; Abdelmotaleb and Saha, 2020). The number of items in each variable, along with the items, has been explained as follows.

#### Socially Responsible Management

Socially responsible management consists of six items, which were adopted by Abdelmotaleb and Saha (2020). The sample items include “my company considers employee social performance in promotions,” “my company considers employee social performance in performance appraisals,” and “my company relates employee social performance to rewards and compensation.”

#### Organizational Morality

Organizational morality consists of three items, which were adopted from Abdelmotaleb and Saha (2020). The sample items include “When I think about my organization, I feel that the name of this organization is (i) honest, (ii) sincere, and (iii) trustworthy.”

#### Employee Vitality

Employee vitality consists of eight items, which were adopted from Carmeli and Spreitzer (2009). The sample items include “I feel active and energetic at work,” “I have high energy to complete my work,” “during the working day I feel I am full of energy,” “I have the energy to successfully do my job,” etc.

#### Employees’ Morale Engagement

Employee’s morale engagement consists of 5 items, which were adopted from Carmeli and Spreitzer (2009). The sample items include “I find the work that I do full of meaning and purpose,” “I am enthusiastic about my job,” “My job inspires me,” “I am proud of the work that I do,” and “To me, my job is challenging.”

#### Employee-Based Brand Equity

Employee-based brand equity consists of 6 items, which were adopted from King and Grace (2010). The sample items include “I really care about the fate of the organization I work for” and “My values are similar to those of the organization I work for.”

### Statistical Tool

This study tested the proposed hypotheses of the study using SEM using Smart PLS 3.3.3. By using this software, path models were developed, and through these path models, the small data sets were analyzed in a short time span (Hair et al., 2017). Smart PLS uses two main models, i.e., the measurement model and the structural model for data analysis.

### Demographic Profile

The demographic profile of the participants in the study is shown in Table 1. Three demographic traits were measured, i.e., age, education, and organizational tenure. It can be observed that 23.87% of participants were aged between 20 and 30 years, 40.97% were aged between 31 and 40 years, 20.97% were aged between 41 and 50 years, and 14.19% were aged above 50 years. Moreover, it can also be observed that 79 participants had a bachelor’s education, and they comprised 25.48% of the total sample. A total of 143 participants had a master’s education, and they made up 46.13% of the entire sample, whereas, 88 individuals had Ph.D. or other qualifications, and they comprised 28.39% of the sample size. Furthermore, 72 participants had an organizational tenure of less than 1 year. A total of 112 had a tenure ranging between 1 and 3 years, 87 had tenure of 4–6 years, and 39 participants had an organizational tenure of more than 6 years.

**TABLE 1:** Demographic analysis.

Demographics	Frequency	Percentage (%)
**Age (years)**		
20–30	74	23.87
31–40	127	40.97
41–50	65	20.97
Above 50	44	14.19
**Education**		
Bachelors	79	25.48
Masters	143	46.13
Ph.D. and others	88	28.39
**Organizational tenure (years)**		
Less than 1	72	23.23
1–3	112	36.13
4–6	87	28.06
More than 6	39	12.58

*N = 310.*

## Data Analysis and Results

### Measurement Model

**FIGURE 2:**
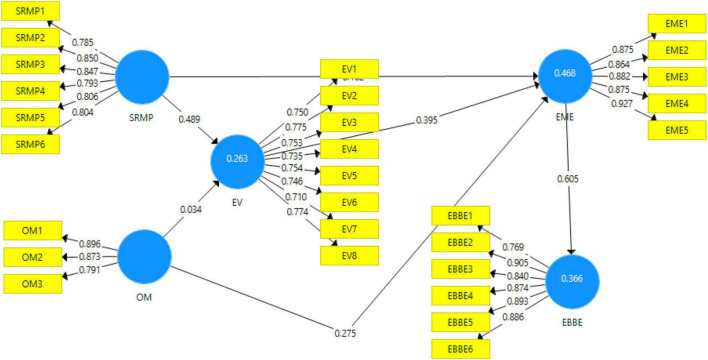
Output of measurement model. SRMP, socially responsible management; OM, organizational morality; EV, employee vitality; EME, employees’ morale engagement; EBBE, employee-based brand equity.

The outcome of the measurement model is depicted in Figure 2. The figure demonstrates the degree to which the predictor variables influence the outcome variables of this study.

The detailed assessment of the measurement model (direct model) is shown in Table 2. It includes the values of factor loadings, variance inflation factor (VIF), Cronbach’s alpha, composite reliability, and average variance extracted (AVE) that were obtained against each of the variables. Jordan and Spiess (2019) posit that the minimum threshold value for factor loading should be above 0.60. The table shows that the values of factor loadings lie between 0.710 and 0.927 thus satisfying this assumption. Moreover, VIF detects collinearity within the proposed model. According to Hair et al. (2014), the outer VIF values should be below 5. It can be observed that all outer VIF values ranged between 1.598 and 4.890. As a result, it can be deduced that there was no collinearity within the proposed model.

**TABLE 2:** Model assessment (direct model).

			Construct reliability and validity		
Factor loadings		VIF	α	Composite reliability	AVE
**Socially responsible management**					
SRMP1	0.785	2.645			
SRMP2	0.850	2.859			
SRMP3	0.747	3.525	0.831	0.846	0.644
SRMP4	0.793	2.408			
SRMP5	0.706	2.166			
SRMP6	0.804	2.262			
**Organizational morality**					
OM1	0.896	2.017			
OM2	0.873	1.986	0.816	0.890	0.730
OM3	0.791	1.598			
**Employee vitality**					
EV1	0.750	1.938			
EV2	0.775	2.751			
EV3	0.753	2.389			
EV4	0.735	1.679			
EV5	0.754	2.763	0.891	0.911	0.562
EV6	0.746	2.708			
EV7	0.710	2.507			
EV8	0.774	3.277			
**Employee moral engagement**					
EME1	0.875	3.109			
EME2	0.764	2.651			
EME3	0.882	3.079	0.830	0.847	0.683
EME4	0.875	2.950			
EME5	0.827	4.890			
**Employee based brand equity**					
EBBE1	0.769	1.812			
EBBE2	0.805	4.033			
EBBE3	0.840	2.669	0.831	0.846	0.644
EBBE4	0.874	3.328			
EBBE5	0.793	3.926			
EBBE6	0.786	3.295			

*SRMP, socially responsible management; OM, organizational morality; EV, employee vitality; EME, employees’ morale engagement; EBBE, employee-based brand equity; VIF, variance inflation factor; α, Cronbach’s alpha; AVE, average variance extracted.*

Table 2 presents the construct reliability and validity. According to Hair et al. (2017), a construct is said to be reliable if the corresponding value of Cronbach’s alpha is above 0.70. Moreover, a value of above 0.70 for composite reliability is regarded as reliable (Peterson and Kim, 2013). The table shows that the values of both of these indicators were above 0.70, which means that the items of the constructs were reliable. Furthermore, the presence of convergent validity was also checked through the values of AVE. According to Dash and Paul (2021), the AVE values should be above 0.50. It can be seen that all values of AVE satisfy this assumption. Convergent validity exists within the proposed model.

The outcome of the tests that were carried out to determine the presence of discriminant validity can be observed in Table 3. The Fornell and Larcker criterion and the Heterotrait–Monotrait (HTMT) ratio are the two major tests that are used to determine the presence of discriminant validity. Discriminant validity tells us whether or not one variable is distinct and unique from another variable. According to Ab Hamid et al. (2017), the value of the HTMT ratio must be lower than 0.90. The table shows that all values of HTMT successfully met this assumption. On the contrary, the underlying assumption for the Fornell and Larcker criterion is that the value at the top of each column should be greater than the values below it (Henseler et al., 2015). It can be seen that this assumption was also met. Hence, it can be deduced that discriminant validity existed within the proposed model.

**TABLE 3:** Discriminant validity.

Fornell–Larcker criterion						Heterotrait–Monotrait ratio					
Constructs	EBBE	EME	EV	OM	SRMP	Constructs	EBBE	EME	EV	OM	SRMP
EBBE	0.863					EBBE					
EME	0.605	0.885				EME	0.646				
EV	0.626	0.581	0.750			EV	0.670	0.612			
OM	0.340	0.538	0.377	0.854		OM	0.386	0.605	0.424		
SRMP	0.466	0.557	0.513	0.703	0.815	SRMP	0.483	0.585	0.530	0.849	

*N = 310.*

*SRMP, socially responsible management; OM, organizational morality; EV, employee vitality; EME, employees’ morale engagement; EBBE, employee-based brand equity.*

The *R*-square values for EBBE, EME, and EV are depicted in Table 4. According to Hair et al. (2017), the sustainability of the model is explained by the *R*-square values, which should be close to 0.50. It can be observed that the *R*-square values for EBBE, EME, and EV are 0.364, 0.462, and 0.258. These values suggest that the proposed model is sustainable. Moreover, the values of *Q*-square can also be seen in Table 4. The *Q*-square values depict the predictive relevance of the proposed model. The values of *Q*-square should be greater than 0 (Hair et al., 2017). All the values of *Q*-square successfully meet this assumption. Therefore, it can be concluded that the proposed model had significant predictive relevance.

**TABLE 4:** *R*-square values for the variables.

	*R*-square	*Q*-square
EBBE	0.364	0.248
EME	0.462	0.341
EV	0.258	0.129
OM		0.108
SRMP		0.112

*N = 310.*

*SRMP, socially responsible management; OM, organizational morality; EV, employee vitality; EME, employees’ morale engagement; EBBE, employee-based brand equity.*

The collinearity statistics are shown in Table 5. Collinearity is examined through the inner VIF values of the constructs. As per Sarstedt et al. (2014), the values of inner VIF should be lower than 5. The table shows that all inner VIF values ranged between 1.000 and 2.299. As a result, it is determined that collinearity was not present in the proposed model.

**TABLE 5:** Collinearity statistics (inner-VIF values).

	EBBE	EME	EV	OM	SRMP
EBBE					
EME	1.000				
EV		1.357			
OM		1.976	1.974		
SRMP		2.299	1.974		

*N = 310.*

*SRMP, socially responsible management; OM, organizational morality; EV, employee vitality; EME, employees’ morale engagement; EBBE, employee-based brand equity.*

### Structural Model

**FIGURE 3:**
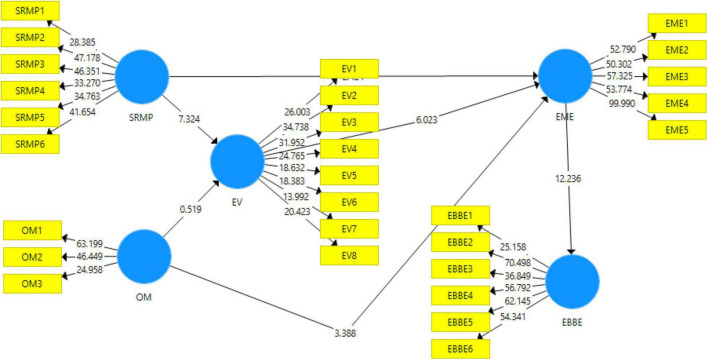
Structural model bootstrapping. SRMP, socially responsible management; OM, organizational morality; EV, employee vitality; EME, employees’ morale engagement; EBBE, employee-based brand equity.

The outcome of the structural model bootstrapping is shown in Figure 3. The structural model includes values of *t*-statistics, and the acceptance/rejection of the proposed hypotheses has been assessed through the PLS-SEM bootstrapping technique. For this purpose, a 95% corrected bootstrap approach was undertaken.

The direct effects of the variables are shown in Table 6. The significance of the result is assessed using the values of *t*-statistics and *p*-values. According to Johnson (2019), the *t*-statistic value should be greater than 1.96. Moreover, Di Leo and Sardanelli (2020) posited that the *p*-value should be below 0.05. Furthermore, the value of *f*^2^ is a depiction of the effect size. Effect size values close to 1 depict higher strength, whereas those close to 0 depict low or weak strength (Meng and Bari, 2019).

**TABLE 6:** Direct effects of the variable.

Paths	H	O	*M*	SD	*t*-Statistics	Effect size (*f*^2^)	*p*-Value	Results
SRMP → EME	H_1_	0.171	0.173	0.070	2.424	0.021	0.016*	*Accepted*
OM → EME	H_2_	0.269	0.270	0.079	3.388	0.072	0.001**	*Accepted*
SRMP → EV	H_3_	0.499	0.499	0.068	7.324	0.164	0.000**	*Accepted*
OM → EV	H_4_	0.044	0.048	0.084	0.519	0.001	0.604	*Rejected*
EME → EBBE	H_7_	0.578	0.579	0.047	12.236	0.576	0.000***	*Accepted*

N = 310, ***p < 0.001, **p < 0.005, *p < 0.05.

*SRMR = 0.085, NFI = 0.753. H, hypothesis; O, original sample; M, sample mean; SD, standard deviation; SRMP, socially responsible management; OM, organizational morality; EV, employee vitality; EME, employees’ morale engagement; EBBE, employee-based brand equity.*

The outcome of the direct relationships is shown in Table 6. H1 stated that SRM had a positive effect on EME. The *t*-statistic value is 2.424, and the *p*-value is 0.016, which suggests that H1 has been accepted. The effect size is 0.021, which depicts weak model strength. H2 predicted that OM had a positive effect on EME. The *t*-statistic value and *p*-value are 3.388 and 0.001, respectively. Hence, H2 has also been accepted. The effect size is 0.072, which indicates weak model strength. H3 proposed that SRM had a positive effect on EV. The value of the *t*-statistic is 7.324, and the *p*-value is 0.000. Therefore, H3 has also been accepted. The effect size is 0.164, which suggests weak model strength. H4 posited that OM had a positive effect on EV. The *t*-statistic value and *p*-value are 0.519 and 0.604, respectively. Therefore, H4 has been rejected. Finally, H7 predicted that EME had a positive effect on employee brand-based equity (EBBE). The *t*-statistic value and *p*-value are 12.236 and 0.000, respectively, which indicate that the results are significant. Therefore, H7 has been accepted. The effect size of 0.576 indicates strong model strength.

The values of standardized root mean square residual (SRMR) and normed fixed index (NFI) are shown in Table 6. SRMR and NFI are indicators that are used to assess the fitness of the proposed model. These values should lie between 0 and 1 (Elsayed and Aneis, 2021). It can be observed that the values of SRMR and NFI were 0.085 and 0.753, respectively. Therefore, it can be ascertained that the proposed model was fit for the data.

The outcome of the indirect effects is shown in Table 7. H5 predicted that EV mediates the relationship between SRM and EME. The *t*-statistic value is 4.537, and the *p*-value is 0.000. Hence, H5 has been accepted. Moreover, H6 posited that EV mediates the relationship between OM and EME. The *t*-statistic value and *p*-value are 0.514 and 0.608, respectively. Hence, H6 has been rejected, and it can be concluded that EV does not mediate the relationship between OM and EME.

**TABLE 7:** Indirect effects of the variable.

Paths	H	O	*M*	SD	*t*-Statistics	*p*-Value	Results
SRMP→ EV→ EME	H_5_	0.197	0.197	0.043	4.537	0.000***	*Accepted*
OM→ EV→ EME	H_6_	0.017	0.020	0.034	0.514	0.608**	*Rejected*

N = 310, ***p < 0.001, **p < 0.005, *p < 0.05.

*H, hypothesis; O, original sample; M, sample mean; SD, standard deviation; SRMP, socially responsible management; OM, organizational morality; EV, employee vitality; EME, employees’ morale engagement; EBBE, employee-based brand equity.*

## Discussion

This research has been conducted from the perspective of female digital labor who can work on different digital platforms for organizations. Certain organizational factors, which are socially and morally connected to organizational management, were studied in this research. For social organizational factors, relationships were evaluated between socially responsible management, EV, and EME, which also had an impact on developing internal brand equity or EBBE. This study also tried to find out the ethical aspects of organizations, such as OM on EV and EME. Moreover, the mediating effects of EV were also tested in this study between social and moral factors of organizational management and EME.

The direct relationship of socially responsible management, which is a type of HRM through CSR, showed significant association with EME and EV. These results indicate that if CSR is fulfilled, and the proper HRM takes place among employees at an organizational level, then it could significantly influence in developing a sense of EME. Previously, some researchers indicated that HRM and CSR are interlinked, and their effects help in boosting organizational productivity and employees’ performance (Shen and Benson, 2016). The possible reason for such results lies in the fact that such management at organizational level has a pulse on employees, and practices are conducted for the employees in a directional way.

It is also obvious that SRM is perceived as CSR, which has an impact on the employees. A few investigations in the recent past have explored the association of perceptions of SRM and the performance of employees (Shen and Benson, 2016). Another similar study concluded that SRM could influence the perceptions of employees about CSR (Shen and Zhang, 2019). Similarly, Shen and Jiuhua Zhu (2011) indicated that SRM had a substantial influence on the commitment of employees to their organizations. All these studies proved the significance of SRM and supported our findings about the role of socially responsible management on EME and employees’ vitality. The other direct relationships between OM, EV, and EME were also studied in this study, and the results indicated that OM had significant association with EME while it could not influence employees’ vitality, which is more of a physical factor associated with employees’ health.

The possible reasoning for such results could be presented as the extent of organizations in providing moral measures at organizational level that are related to the well-being of employees. These moral values are conferred upon the functioning of organizations, and employees, in return, feel morally associated with their respective organizations. It is supported by the fact that integrity, honesty, and reliability are the pre-requisites of the functioning at workplaces. All these are referred to as OM (Abdelmotaleb and Saha, 2020). Previous research has focused on morality as a collective attribute (Shadnam et al., 2020), and those collective attributes showed positive associations of OM with employees’ engagement at workplaces. The association between OM and EV could not prove its worth as vitality indicators are more affiliated with the health of employees and OM had no impact on health-related aspects of employees.

The indirect effects of EV were also tested in this study between socially responsible management, OM, and EME. The relationships between SRM were mediated by the EV, and it helped in boosting EME. This is possible due to the reason that such human resource-based practices influence the behaviors of employees positively. Previously, some of the HRM practices were indicated to be necessary for the vitality of the employees, which led the employees to work efficiently in teams (Scheppingen et al., 2014; Tummers et al., 2018). This kind of performance is required at organizational levels. Therefore, this study shows another level of employees’ bond with their organizations. The indirect effects of employees’ vitality could not prove their aiding role between OM and EME to their organizations.

This indicates that EV is not related to the moral practices of organizations, and it could also not provide any mediation between OM and EME. Previously, Tummers et al. (2018) looked at how work factors affect EV. These job features focused only on knowledge management, its transfusions, and work-related engagement, and they had an association with employees’ vitality. The direct effects of EME showed that it had a significant association with EBBE. As indicated by Sendawula et al. (2018), employees’ engagement is related to certain factors at workplaces, which could boost their performance or demoralize them at work. Once employees are morally engaged with their organizations, they could work for the branding of their organizations. It was also supported by Gupta and Shaheen (2018) and Boukis and Christodoulides (2020) stated that morally engaged employees have the potential to develop EBBE.

### Theoretical Contribution

This study contributes to the environmental and HRM literature. First, this study examined the role of organization-level variables (such as organizational mobility and socially responsible management) on employee behavior, which has not been analyzed earlier. The existing literature is also enriched by adding a new mediating variable, i.e., EV, in the relationship between socially responsible management and EME and also between organizational mobility and EME. New findings were found between EME and EBBE, which added value to the existing literature. Fewer studies were conducted to examine the relationship between these variables only among female employees, so this study enriched the literature in this regard.

### Practical Implications

This study provides several practical implications for HR managers and practitioners who are looking for strategies to enhance social responsibility management and OM within the organization. CSR programs are developed to address the concerns and interests of the external stakeholders; however, these programs do not incorporate the interests of the internal employees. On the basis of the findings obtained from this study, it is significant for organizations to adopt and implement socially responsible management practices, as these practices increase EV, which further boosts both EME and EBBE. Moreover, organizations should put effort in providing CSR training to encourage CSR as a significant organizational value. Human resource managers must conduct performance appraisals to measure the social performance of their employees. Additionally, HR managers must provide promotions and increments to employees who have demonstrated positive social performance. In addition to this, policies must be devised by the management of the organization so that social responsibility can be increased within the organization. When employees feel that OM is high, the level of EME will be enhanced, thus increasing the success of the organization.

### Limitations and Future Directions

In addition to the implications explained above, this study also presents a few limitations, which could be used in future research. This study used a small sample size; therefore, future studies can conduct the study using a larger sample size for generalizing the data to the entire population. Moreover, the present study was conducted on female employees working in software houses from home, thus, future studies can be conducted on both genders, i.e., males and females, in order to examine the results on the male population as well. Future studies can also be conducted on other industries, for example, the banking or hospitality sector. Furthermore, studies can be conducted on other Asian countries or western regions. Another limitation of the study is related to the study variables. This study examined the role of socially responsible management and OM on EME with the mediation of EV. Also, the study examined the role of EME on EBBE. Future studies can use other variables, such as organizational justice, to examine the role of this variable in the present framework.

## Conclusion

Human resource management plays a significant role in achieving a competitive advantage. HRM devises policies for the employees so that they can effectively perform in the organization. Therefore, this study examined the role of socially responsible management and OM on EME with the mediation of EV. Also, the study examined the role of EME on EBBE. The investigation was conducted on female employees working in software houses from home in China. The study found that socially responsible management has an effect on both EME and EV. The study revealed that OM has an effect on EME, and EME has an impact on EBBE. However, an insignificant relationship was found between OM and EV. The results of the mediation analysis showed that EV mediates the relationship between socially responsible management and EME. However, EV did not mediate the relationship between OM and EME.

## Data Availability Statement

The original contributions presented in the study are included in the article/supplementary material, further inquiries can be directed to the corresponding author.

## Ethics Statement

The studies involving human participants were reviewed and approved by Beijing Language and Culture University, China. The patients/participants provided their written informed consent to participate in this study. The study was conducted in accordance with the Declaration of Helsinki.

## Author Contributions

SH: conceptualization, data collection, and writing the draft.

## Conflict of Interest

The author declares that the research was conducted in the absence of any commercial or financial relationships that could be construed as a potential conflict of interest.

## Publisher’s Note

All claims expressed in this article are solely those of the authors and do not necessarily represent those of their affiliated organizations, or those of the publisher, the editors and the reviewers. Any product that may be evaluated in this article, or claim that may be made by its manufacturer, is not guaranteed or endorsed by the publisher.
